# Influence of the Weak Nuclear Force on Metal-Promoted Autocatalytic Strecker Synthesis of Amino Acids: Formation of a Chiral Pool of Precursors for Prebiotic Peptide and Protein Synthesis

**DOI:** 10.3390/life14010066

**Published:** 2023-12-30

**Authors:** J. A. Cowan

**Affiliations:** Department of Chemistry and Biochemistry, The Ohio State University, 100 West 18th Avenue, Columbus, OH 43210, USA; cowan.2@osu.edu

**Keywords:** parity non-conserving, autocatalysis, amino acid, chirality, weak nuclear force, alkaline earth

## Abstract

Natural chiral amino acids typically adopt an L structural configuration. While a preference for specific molecular chiralities is observed throughout biology and cellular chemistry, the origins of this preference are unclear. In a previous report the origin of enantiomeric selectivity was analyzed in terms of an “RNA World” model, and a pathway to a chiral preference for d-ribose was proposed based on the autocatalytic transformation of glyceraldehyde as a precursor to the formation of sugars. Metal-ion-promoted catalysis allows the parity non-conserving (PNC) weak nuclear interaction to influence the chirality of a nascent chiral carbon center. Since the PNC effect is the only natural property with an inherent handedness, it is an obvious candidate to influence enantiomeric preference from a catalytic reaction performed over geologically relevant time scales. The PNC influence requires and emphasizes the important role of catalytic metal ions in primordial chemistry. In this study, the impact of geologically available divalent calcium and higher Z alkaline earth elements are examined as mediators of chiral preference. Detailed calculations of the magnitude of the effect are presented, including the influence of time, temperature, pH, and metal ion identity. It is concluded that metal ions can direct chiral preference for amino acid synthesis via a metal-promoted autocatalytic Strecker reaction within a relatively short geological timeframe, thereby providing a pool of l-amino acids for catalytic chemistry evolving either from an RNA-world model of molecular evolution or alternative pathways to protein synthesis.

## 1. Introduction

**Figure 1:**
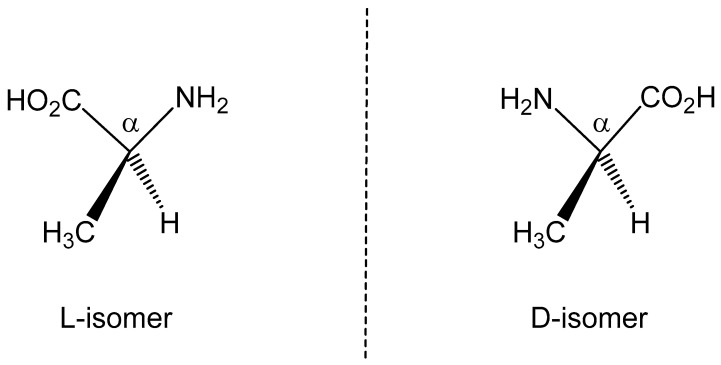
Molecular chirality represented by the two mirror-image enantiomers of the amino acid alanine, showing the chiral carbon center designated by α.

Previous work has described an RNA-centered evolutionary pathway to the chiral preference exhibited by the molecules of life [1]. Specifically, the origin of enantiomeric selectivity was considered within the context of an “RNA World” model and in terms of a mechanism involving autocatalytic reaction of glyceraldehyde on a reaction path that results in the formation of saccharides relevant to the synthesis of d-ribose [1]. While an alternative [GADV]-protein world hypothesis has been proposed [2], the RNA world model has received more general acceptance. Nevertheless, the synthesis of amino acids is a requisite step in evolutionary development. Natural chiral amino acids adopt an l-configuration (exemplified by alanine in Figure 1). Such a chiral preference could naturally emerge from RNA-derived catalytic chemistry if chirality were already imprinted through a parity non-conserving (PNC) effect [1]. However, it is also possible that PNC-directed catalytic chemistry could establish a pool of l-amino acids available for use in primordial RNA-promoted peptide or protein synthesis. Other reports have described alternative pathways to amino acid enantiomeric preference, spanning the range from a synthetic pathway based on chiral ribose-catalyzed synthesis of chiral amino acids [3] to the influence of magnetic fields on the nitrogen nuclear spin [4]. An overview of the extensive literature on the topic of chiral selection through spontaneous symmetry breaking and seeding mechanisms from extraterrestrial meteorites and comets, as well as alternative mechanisms, is presented in [1].

**Figure 2:**
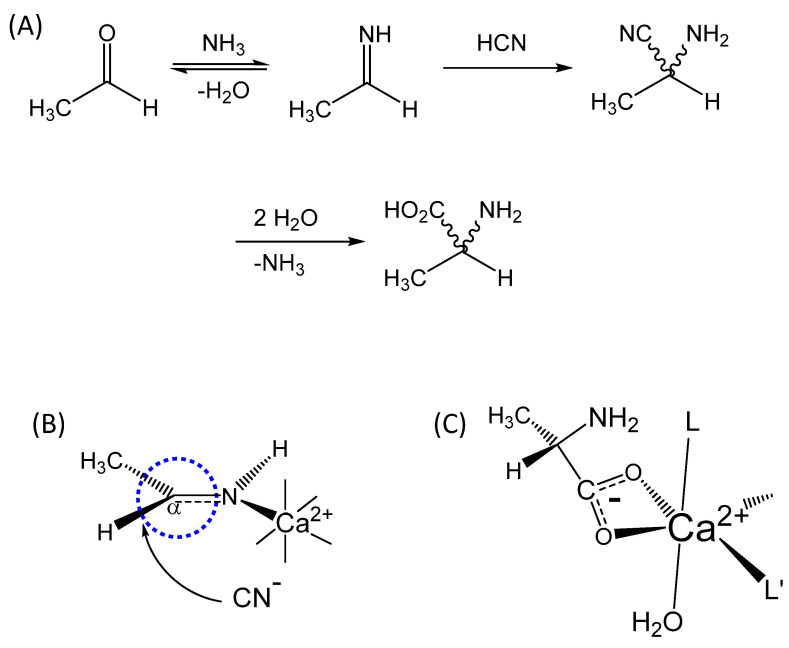
(**A**) Alanine synthesis using a Strecker mechanism, where the wiggly bonds indicate indeterminate stereochemistry. (**B**) Illustrating a calcium-promoted step on the reaction pathway where a PNC effect from Ca^2+^ could influence chiral preference at the C_α_ carbon (highlighted by circling in dashed blue). (**C**) An example of an Ala-bound calcium complex in a metal-mediated autocatalytic pathway, where L and L′ could be water, a bifurcating carboxylate from another amino acid [5], or other ligands.

This manuscript presents a theoretical analysis of an autocatalytic reaction involving a metal-mediated Strecker synthesis (Figure 2) of natural amino acids that demonstrates a chiral preference based on metal-induced PNC effects. This affords an observable enantiomeric preference within very short geological timeframes (as short as a few decades) that could provide a pool of chiral amino acid precursors for use in peptide and protein synthesis.

**Figure 3:**
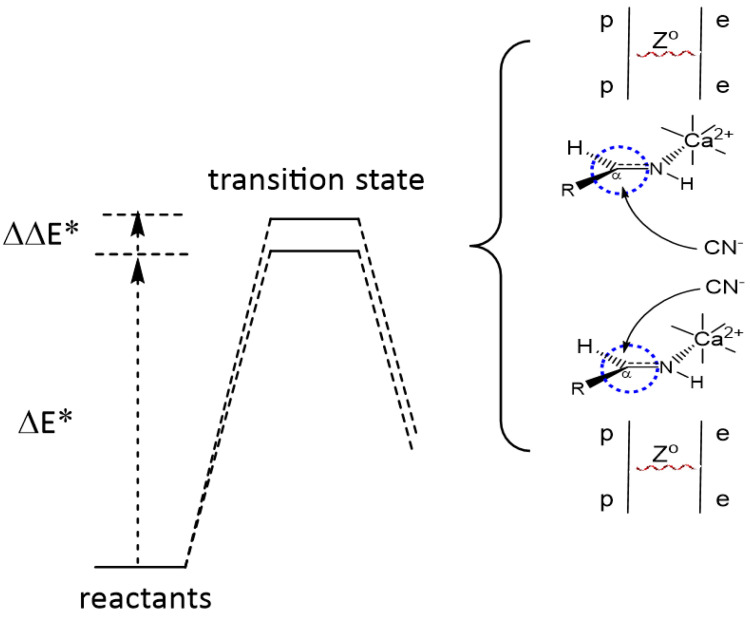
Activation barriers for two enantiomeric transition states (the first one is shown as ΔE*), where ΔΔE* = ΔE^PNC^ represents the difference in activation energies between the two enantiomers arising from the influence of PNC. The calcium-promoted reaction proceeds through two enantiomeric transition states that have slightly different energies as a result of the inherent chirality of the weak nuclear force from the influence of the divalent metal cofactor. As shown in the Feynman diagrams (right), Z° bosons mediate the weak current interaction from protons (p) on the metal ion to electrons (e) at the C_α_ carbon (circled in blue). In this image, R represents one of the standard amino acid sidechains.

Discrimination in the formation of the two enantiomeric forms arises from the distinct activation energies that reflect diastereomeric selection in the transition state (Figure 3). While the difference in activation energies (ΔΔE*) for this reaction is extremely small, in the context of an autocatalytic reaction that is repeated multiple times, and in particular over evolutionary timescales, the enhancement of one enantiomer over another will be significant and is reflected by Equation (1), where n is the number of catalytic turnovers within the time period under consideration. Catalytic chemistry is suggested to be promoted via divalent calcium, an element that would have been particularly prevalent in primitive clays and minerals (Table 1) [6,7].

(1) $$k^{enhance}={\left(exp(∆∆E^{*}/RT)\right)}^{n}$$

Metal-promoted Strecker reactions are well documented in the literature [8,9,10,11,12,13,14], and amino acids have been shown to coordinate with alkaline earth by a bifurcating carboxylate, as shown in Figure 2C [5]. Potentiometric estimates of binding affinity indicate stability constant log_10_β~10 for glycine [15], where typically alanine has a slightly higher affinity [16,17], and so such interactions are certainly relevant to primordial chemistry and catalysis. It is possible that more than one amino acid coordinates to the catalytic metal; however, in this report, it is assumed that only one amino acid ligand is bound. Additional amino acid ligands would simply amplify any additional chiral inductive influence through diastereomeric discrimination of a pre-existing chiral center. This latter effect is not directly pertinent to the hypothesis put forward in this study but is relevant to some aspects of the later discussion section, and the practical details of the chemistry, and is addressed later in the manuscript. 

**Figure 4:**
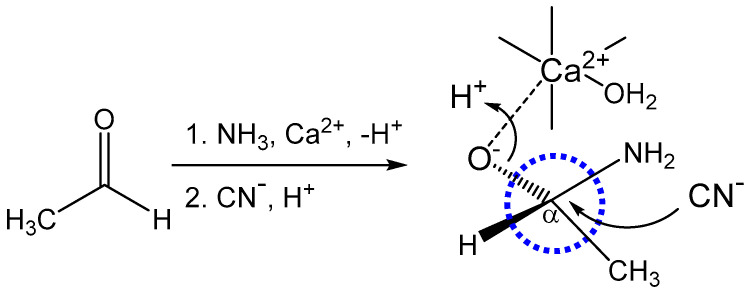
A plausible catalytic intermediate formed en route to imine formation may be intercepted by an oxophilic alkaline earth ion. The transiently-bound calcium ion may be stabilized by direct interaction with the amine or, more likely, through H-bonding to a calcium-bound water. Nucleophilic attack by CN^-^ in the second step occurs from above the plane of the page, and the oxygen center is protonated as it is released. The nascent chiral C_α_ carbon is circled in blue.

While metal-promoted Strecker reactions are recognized, the mechanistic involvement of the metal catalyst is less clear. While action through imine coordination (Figure 2) is often considered, binding to an oxygen intermediate is plausible (especially if chelated via nitrogen and oxygen (Figure 4) and is also considered in calculations of the PNC effect detailed in this work.

Herein, it is shown that a PNC mechanism meets the energy limits for the selection of a specific enantiomer by an autocatalytic reaction when the reaction involves alkaline earth metal ions and occurs even over short geological timeframes. The assumptions made are conservative and provide a lower benchmark estimate for the magnitude of ΔE^PNC^. 

## 2. Materials and Methods

Hergstrom et al. were among the early proponents of a key role for the weak nuclear force and parity non-conservation (PNC) in chiral discrimination within molecular systems [18,19]. Significantly, these and other investigations of the influence of the weak nuclear interaction on atomic and molecular species were very much dependent on the pioneering theoretical framework established by Bouchiat and Bouchiat [20]. The studies by Hergstrom and others built on this theoretical foundation and provided an important benchmark for practical application to specific molecular species. Importantly, their work highlighted the “single-center” problem that requires two matrix elements that are centered on distinct atoms if an observable effect is to be realized [18,19]. Ideally, the two atomic centers are in close proximity, as illustrated in Figure 2 and Figure 4, which are even closer than the recently considered aldol reaction [1].

The catalytic and chiral centers are different for a metal-promoted Strecker reaction and are not directly connected by covalent bonds. As a result, the calculation focuses on the carbon-centered orbitals that directly overlap with the nucleus of an alkaline earth ion (Ca^2+^, Sr^2+^, or Ba^2+^) [1]. This permits a significant simplification of calculations using Equation (2),

(2) $$\Delta E^{PNC}=\frac{G\alpha }{\sqrt{2}}\frac{Q_{\omega }\left(P\right)\left(\Delta \right)}{E_{a}-E_{b}}$$

where ΔE^PNC^ represents the second-order perturbation term for the PNC-induced energy splitting*, α* is the fine structure constant, *G* is the Fermi constant, *Q_ω_* is the weak charge (Equation (3)), (*Δ*) is the spin-orbit coupling term that provides a first-order correction to the wavefunctions used in the calculations, (*P*) is the PNC matrix element, and *E_a_ − E_b_* is the energy difference in the spin-orbit coupling term for ground and higher energy wavefunctions. Spin-orbit coupling is required in a non-relativistic approach because the coordinate part of Equation (2) is only imaginary, so an imaginary term from spin-orbit coupling is required to yield an observable outcome.

Q_w_ = (1 – 4sin^2^θ_ω_) − N (3)

Bouchiat and Bouchiat, and also Hergstrom et al., have shown that in the case of a single-center problem, there is a Z^5^ dependence on ΔE^PNC^, where Z is the atomic number [18,19], representing the “heavy atom effect”. However, in the case of a single-center, the magnitude of ΔE^PNC^ is theoretically zero because a Dirac δ function embedded in Equation (2) [18,19] has matrix elements that involve only s and p states, while the spin-orbit matrix elements yield a non-zero value only if an s state is not involved [19]. In this work, the two relevant atomic centers are the carbon (C_α_) center and the alkaline earth metal cation (M). The (P) term was evaluated for M^2+^ and shows a (Z)^4^ dependence, and the Q_w_ component has a (Z) dependence, providing an overall (Z)^5^ dependence for ΔE^PNC^.

The energy difference that results from the influence of the weak interaction at carbon C_α_ (Figure 2) is defined by Equation (2) [18,19]. The PNC matrix element is represented by Equation (4) [18,19],

(4) $$\left(P\right)=\frac{i\;\sqrt{3}}{4\pi }\;R_{ns}\left(0\right)\frac{dR_{n^{’}p}\left(r\right)}{dr}\;{|}_{r=0}$$

where *R_ns_* and *R_n_*_′*p*_ are the radial wavefunctions for the atomic orbitals on the metal cation.

The spin-orbit coupling matrix element for carbon is diagonal in the *np* orbitals used and was calculated for the high energy *n*′′*p* orbital by direct computation using Equation (5), where *f*(*r*) is a function of distance (in this case, it is 1/*r*^3^) [21], which mixes the *p* orbitals on the developing chiral C_α_ carbon.

(5) $$\left(\Delta \right)=<n’’p_{x}|f(r)l_{y}|2p_{z}>$$

## 3. Results

In the context of PNC, catalysis by divalent calcium and other alkaline-earth elements addresses several problems that have hindered prior attempts to experimentally measure PNC effects [22,23,24,25]. Calculations are initially applied to the most abundant alkaline earth element in the earth’s crust, namely calcium (Table 1), and then to strontium and barium. Two simplifications that contrast with prior work were used to perform these calculations [26,27]. First, the single-center problem is resolved by considering two centers: an alkaline earth cation (Ca^2+^, Sr^2+^, Ba^2+^) and a separate carbon center. Second, the through-space electrostatic interaction between the metal cation and the nascent chiral carbon allows the problem to be addressed using atomic orbitals on calcium and carbon since there is no significant covalency or electron sharing between each.

When defining the matrix elements, and in the representative case of a Ca^2+^ ion separated from the carbon center of interest (C_α_) by a conservative distance of 5.73 au, 6.07 au for Sr^2+^, and 6.39 au for Ba^2+^, representing the sum of the metal-imine bonds, the calculations around this linear geometry provide conservative limits on estimates of the magnitude of the effect over longer timeframes (Table 2). This linear geometry was used only to allow comparison with prior conservative calculations on a different chemical system [1]. A more realistic set of internuclear distance constraints based on a trigonal planar 120° angular geometry for the Ca-N-C_α_ bonding arrangement (Figure 2) yielded values of 4.98 au for Ca^2+^, 5.29 au for Sr^2+^, and 5.58 au for Ba^2+^ (results of calculations are also summarized in Table 2). As previously discussed [1], the volume element included the entire ion since the weak nuclear force is communicated to the nascent chiral C_α_ carbon through an electronic interaction. In as much as the transition state will actually proceed through an approximately tetrahedral species, the actual ΔE^PNC^ values will fall between the two limits of angular and linear approximations.

Calculations were performed using a spherical polar coordinate system and considered volume elements for carbon p orbitals that incorporated a sphere around the divalent ion defined by the ionic radius (Ca^2+^ 2.16 au, Sr^2+^ 2.50 au, and Ba^2+^ 2.82 au) and an intercenter distance that was defined by triangulation of the bond distances (covalent -C=NH of 2.44 au, and dative Ca^2+^-NH= of 3.39 au, Figure 2) from the prochiral carbon (C_α_) to the catalytic metal ion. Boundary limits for the θ and φ coordinates were determined from the geometric constraints of the metal to C_α_ distance and the ionic radii of the metal ions. 

Values for various parameters defined in Equations (2)–(5) were determined as before [1]. The 1s orbital, which lies closest to the atomic nucleus, was mixed with the most stable empty p orbital (4p for the example of calcium), so R_1s_(0) = 2.(Z_Ca_)^3/2^. Using Z_eff_ = 20 for calcium, the electronic contribution for the 1s orbital yielded dR_4p_(r)/dr|_r=0_ = (10.(Z_Ca_)^5/2^)/32√15, and the sin^2^θ_w_ = 0.25 term in Equation (3) yielded Q_w_^Ca^ = −20 for _20_Ca [1]. With the defined distance separating the prochiral C_α_ atom and the metal cation, the carbon 6p orbital yielded the largest contribution to the spin-orbit coupling term (Δ). As an estimate of the *E_a_ − E_b_* term in the denominator of Equation (2), a conservative value of 0.414 au was used, which corresponds to the ionization energy for a carbon center [28,29,30]. This provided an upper boundary for the energy difference between the carbon 2p and higher energy np orbitals and provided a conservative estimate of the ΔE^PNC^ Values. Table 2 summarizes the ΔE^PNC^ values determined from Equations (2)–(5) for each divalent cation relative to the C_α_ carbon, and Table 3, Table 4 and Table 5 illustrate how those ΔE^PNC^ values translate into enantiomeric selectivities over various time frames for varying assumptions of t_1/2_ for the Strecker reaction. For comparison, results from prior work on metal-promoted aldol chemistry, which conservatively assumed a linear geometry, are summarized in the last column of Table 2. The shorter distance, reflecting the loss of one atom and bond, is clearly evident with a factor of 10^7^ difference in magnitude for the PNC effect.

Calculations were also performed in the case of plausible metal binding to the oxygen center in the hemiaminal reaction intermediate prior to imine formation (Figure 4). Again, these were carried out with the initial assumption of a very conservative linear arrangement of atomic centers between the metal ion and C_α_ carbon, with distances of 6.60 au for Ca^2+^, 6.94 au for Sr^2+^, and 7.26 au for Ba^2+^. The results of calculations around this linear geometry are summarized in Table 6 and compared with the results from a prior aldol study [1]. A more realistic set of internuclear distance constraints, based on a tetrahedral 109.5° angular geometry for the Ca-O-C_α_ bonding arrangement [31] and typical ionic and covalent bond distances, yielded values of 5.43 au for Ca^2+^, 5.73 au for Sr^2+^, and 6.02 au for Ba^2+^ (results of calculations are also summarized in Table 6). Again, the volume element included the entire metal cation, and calculations were performed using spherical polar coordinates and considered a volume element for the carbon atomic orbitals that incorporated a sphere around the divalent ion, as described earlier. Values for various parameters defined in Equations (2)–(5) were also determined as described earlier and are summarized in Table 6. Table 3, Table 4 and Table 5 illustrate how those ΔE^PNC^ values translate into enantiomeric selectivities over various time frames for varying assumptions of t_1/2_. As described earlier, the actual transition state will proceed through a distinct transient geometry (an approximate trigonal bipyramidal species expected for an S_N_2 substitution path) and the ΔE^PNC^ values for such will fall between the two limits of angular and linear approximations detailed in Table 6. These therefore provide a conservative basis for discussion.

The influence of reaction rate constants (or reaction halftimes, t_1/2_) was also examined and is summarized in Table 3, Table 4 and Table 5. The rate-limiting step for the Strecker synthesis of amino acids is the final hydrolysis of the nitrile functionality to a carboxylic acid [32]. Under prebiotic conditions, with a pH of 8 and a temperature of 25 °C, a rate constant of 5.2 × 10^−6^ h^−1^ has been reported, corresponding to a t_1/2_ of 15.2 years [32]. However, the reaction is substantially catalyzed by formaldehyde, which is relatively abundant on primordial earth with a lower limit concentration of 0.02 M [32], and would subsequently yield lower limits for the observed rates that are on the order of 1.2 × 10^3^ greater [32]. That is minimal rate constants of 0.37 min^−1^ (t_½_~1.8 min) or faster. This indicates that Table 4 provides the most relevant estimates of chiral preference based on ΔE^PNC^ effects.

A temperature of 315 K was taken in these calculations as representative of the approximate average ocean temperature on early Earth [1]. This was previously estimated from O/Si isotope ratios and the melting temperatures of ribosomal nucleic acid sequences from ancient archaebacteria [33,34,35]. Given the higher and lower limits, a slightly smaller enhancement may be expected at higher temperatures but is offset by the more rapid catalytic turnover at the elevated temperature, and vice versa. A temperature difference within a range relevant to early earth chemistry does not significantly change the results or conclusions. The early ocean pH is also relevant and is considered as slightly alkaline (~7.3–7.7) [36], which is favorable for metal-promoted Strecker reactions. 

## 4. Discussion

Parity non-conservation has previously been invoked as a possible pathway to chiral discrimination in proteins and nucleic acids; however, the magnitude of the effect was found to be too small for direct observation [22,23,24,25]. An intrinsic ΔE^PNC^ of ~10^−20^ au has been predicted for the discrimination of amino acid enantiomers [37]. By contrast, a recent study described several factors (autocatalysis, a two-center approach, metal mediation, and viable chemical mechanisms) that could enhance the PNC contribution and frame the process in terms of viable chemistry [1]. In that study, the chirality of ribose was addressed in the context of the RNA world model and illustrated the viability of PNC as a possible promoter of a selected molecular chirality in nucleic acids and proteins, as well as a reasonable chemical mechanism to incorporate PNC influences into molecular models of biological evolution. This also requires realistic chemical reactions that facilitate the selection in a manner that can become manifest by natural, synthetic pathways over geological timescales. 

In this study, it is shown that a metal-mediated Strecker synthesis provides a plausible reaction mechanism for the production of a chiral amino acid pool. The Strecker reaction has only one reversible step, namely the initial formation of an imine following the reaction of ammonia with the carbonyl group (Figure 2A). The subsequent nucleophilic attack by cyanide is the reaction step that creates the chiral C_α_ carbon (Figure 2B and Figure 4), and that step is not reversible over the reaction timeframes described herein. Consequently, the chiral center that is formed is stereochemically stable. Furthermore, the C_α_ hydrogen is also non-acidic, lying adjacent to an electronegative nitrogen center, so there is no way to scramble the stereochemistry through enolization chemistry. Indeed, the stereochemical instability of amino acids would undermine the dominance of preferred stereoisomers for amino acids, peptides, and proteins.

In the context of chiral discrimination through multiple turnovers in the autocatalytic cycle, we can amplify and clarify the mechanism of discrimination by considering additional details of the metal-catalyzed reaction. It was noted earlier that amino acids coordinate with alkaline earth cations in a bifurcating (chelating) manner, as illustrated in Figure 2C. When metal ions bind chiral chelating ligands, all bound ligands tend to have the same chiral form to prevent unfavorable steric clashes. Consequently, an L-form of alanine bound to a metal will prefer to catalyze the formation of a new L-alanine, and an R-form bound to a metal will prefer to catalyze the formation of a new R-form. However, the influence of the weak nuclear interaction favors the formation of one chiral form over another and the enrichment of that form over long periods of time. This is the molecular pathway by which autocatalytic chemistry provides a pathway to enantiomeric selectivity in the synthesis of amino acids.

Herein, the PNC influence has been assessed from the viewpoint of catalysis via a more traditional metal-imine complex (Figure 2B) and a more novel but, in some regards, more plausible metal–oxygen intermediate (Figure 4). The former affords a modestly better outcome because of the shorter -C=NH imine bond (compare Table 2 and Table 6). But, either path provides a vehicle for selectivity in amino acid synthesis. This pool of chiral amino acids could be used either in the context of a peptide- or protein-based evolutionary model [2] or the currently more attractive RNA model, which is the most viable driver of prebiotic chemistry and molecular synthesis. In either context, the reaction profiles described herein demonstrate that alternative pathways are available for the production of pools of chiral building blocks, such as amino acids. Given that metal-catalyzed aldol condensation reactions are not the only reaction schemes by which chiral selection may arise [1], this work demonstrates a viable mechanism for the formation of the chiral amino acid building blocks of proteins and other cellular molecules, based on the parity-violating influence of the weak nuclear force. It fully complements other published reports that describe alternative physical and chemical mechanisms to generate chiral centers in the molecules of life [4,26,38,39,40,41,42,43,44]. 

As noted earlier, prior solution kinetic studies of the Strecker reaction under prebiotic conditions yielded limiting rate constants on the order of a few min^−1^ [32]. By use of Equation (1) and the ΔΔE* calculated from the PNC energy difference for the alkaline earth ions summarized in Table 2 and Table 6, enantiomeric selectivity was determined over the stated time periods using Equation (1) and values are summarized in Table 3, Table 4 and Table 5. These values reflect the enantiomeric ratio that could be achieved over a range of ΔE^PNC^ values and timeframes with a fixed reaction rate constant (or half reaction time) and also with a fixed ΔE^PNC^ but variable rate constant and time frames. Assuming a reaction rate corresponding to a t_1/2_ of ~1 min (the half-time most consistent with the reactivity described earlier)^,^ chiral discrimination is evident within evolutionary time frames when cellular life is first thought to develop [45] and even over only 10 to 10^3^ years. 

It is instructive to compare the results of other published studies of the influence of PNC on enantiomeric selectivity to gauge the relative magnitudes of the effect and the range of structural forms that chiral discrimination might impact. In the early work of Hergstrom and coworkers [18], the molecule ethylene constituted the molecular system under investigation, which contains a H_2_C=CH_2_ double bond and where chirality stems from a twisting of the two CH_2_ fragments relative to each other. No heavy atoms were involved, and the study employed a molecular orbital (MO) approach, which is appropriate given that the bonding is covalent. The absence of heavy atoms and the MO treatment that diminishes the PNC influence due to extensive delocalization of electron density contributed to the relatively small calculated ΔE^PNC^.

In a related study, Quack and coworkers [46] focused on a different molecule, hydrogen peroxide (HO-OH). This molecule has free rotation around the O-O bond and again lacks a heavy atom, although the atomic number of oxygen is 8 and exceeds that of carbon, which is 6. The difference in atomic numbers can rationalize the difference (10^−20^ au versus 10^−19^ au in the Hergstrom and Quack studies, respectively). The more sophisticated calculation in the Quack study most likely also contributed to the difference in magnitude of the calculated ΔE^PNC^ values.

In another study by Mirzaeva and Kozlova [47], the molecules under consideration were very different from those examined by Hergstrom and Quack. For this investigation, a two-metal system bridged by a DABCO ligand was studied. Again, a molecular orbital treatment was used. The data reported for ΔE^PNC^ for a series of group 10 elements (Zn^2+^, Cd^2+,^ and Hg^2+^) yielded values ranging from 10^−15^ to 10^−18^ au (corresponding to the values of 10^−12^ to 10^−15^ kJ/mole in the units used in the study). The smaller magnitude of the calculated ΔE^PNC^ is expected for such a large molecule with many bonds communicating the effect. Significantly, the ΔE^PNC^ values increased with an increasing atomic number of the metals examined. It is also important to note that the molecules under consideration by Hergstrom, Quack, and Mirzaeva all attain chirality through a twisted molecular shape. In the case of the amino acid described in this work, chirality arises at an atomic center, and so this also differentiates these prior reports from what is described herein.

As described in earlier work [1], reaction temperature must also be considered as a factor influencing kinetic activation barriers but does not impact the results or conclusions for the reasons stated earlier, given the modest variation and counterbalancing influence of the parameters. Early ocean pH is also relevant and is recognized to be modestly alkaline [36]. As such, it would not impact the results presented and, in fact, is consistent with the proposed reaction conditions [1].

While calcium ion is the most prevalent (Table 1) and most probable metal cation to mediate catalytic activity, and is abundantly found in primordial clays and minerals, heavier alkaline earth elements that are also found in the lithosphere (strontium and barium) should also be considered since these will show an enhanced Z- effect. Calculations for each cation show the expected significant increase in the magnitude for ΔE^PNC^**,** and therefore ΔΔE*, arising from the influence of the weak nuclear interaction (Table 2 and Table 6) from the increase in Z and Q_W_. This results in a much shorter timeframe for the influence on enantiomeric selectivity to be potentially observed (see Table 4 in particular). Both strontium and barium ions occur in relatively high concentrations in the Earth’s crust (Table 1) and are more soluble than calcium. They are also found in many extant calcium minerals, such as barytocalcite, BaCa(CO_3_)_2_, and olekminskite, Sr(Sr,Ca,Ba)(CO_3_)_2_, as well as their exclusive mineral forms.

In summary, it is herein shown that consideration of metal-promoted catalysis and autocatalytic reactions can account for the selective formation of chiral amino acid building blocks for either peptide or protein formation. This builds into, and is consistent with, an RNA world model over evolutionary timeframes [1] and is fully consistent with prior conclusions that chiral preference, in the case of amino acids, could have arisen from distinct chemical mechanisms [3,4,26,38,39,40,41,42,43,44]. A reviewer suggested a reasonable alternative selection pathway involving transient binding of soluble chiral amino acids through preferential binding to a mineral lattice of alkaline earth ions that could also create an imbalance of enantiomeric enrichment in a solution phase. Such discrimination would arise through PNC-derived differences in ground state (binding) energies for each enantiomeric form, and is worthy of future analysis and comparison with transition state models.

## Figures and Tables

**Table 1:** Approximate relative abundance of the elements in the Earth’s crust, relative to 10^6^ atoms of silicon (from an illustration in reference [7]).

Cation	Relative Abundance
Ca^2+^	8 × 10^4^
Sr^2+^	2 × 10^2^
Ba^2+^	10^2^

**Table 2:** Calculated ΔE^PNC^ values based on a metal-catalyzed Strecker reaction via a trans-coordinated imine intermediate. ΔE^PNC^ values determined for single Ca^2+^, Sr^2+^, and Ba^2+^ centers, accounting for variations in orbital energies and atomic numbers. For Ca^2+^, Sr^2+^, and Ba^2+^, n′ = 4 to 8 orbital contributions, respectively, were considered for the parity term (P) in Equation (4), and the relative contributions from n′′ = 3 to 12 accounted for in the carbon spin-orbit coupling term (Δ) in Equation (5). Both conservative linear and angular arrangements are represented.

Cation	ΔE^PNC^ (au) (Angular)	ΔE^PNC^ (au) (Linear)	ΔE^PNC^ (au) (Linear) ALDOL [1]
_20_Ca^2+^	5.4 × 10^−12^	5.8 × 10^−13^	0.3 × 10^−20^
_38_Sr^2+^	1.2 × 10^−10^	1.2 × 10^−12^	0.6 × 10^−19^
_56_Ba^2+^	6.7 × 10^−10^	6.6 × 10^−11^	0.3 × 10^−18^

**Table 3:** Dependence of enantiomeric selectivities on ΔE^PNC^ for t_1/2_~one second.

ΔE^PNC^ (au)	Selectivity Factor over 10^4^ Years	Selectivity Factor over 10^3^ Years	Selectivity Factor over 10^2^ Years	Selectivity Factor over 10 Years	Selectivity Factor over 1 Year	Selectivity Factor over 0.1 Year
10^−13^	2.6 × 10 ^14^	27.6	1.39	1.03	1	1
10^−12^	1.4 × 10^144^	2.6 × 10 ^14^	27.6	1.39	1.03	1
10^−11^	>10^300^	1.4 × 10^144^	2.6 × 10^14^	27.6	1.39	1.03
10^−10^	>10^300^	>10^300^	1.4 × 10^144^	2.6 × 10^14^	27.6	1.39
10^−9^	>10^300^	>10^300^	>10^300^	1.4 × 10^144^	2.6 × 10 ^14^	27.6

**Table 4:** Dependence of enantiomeric selectivities on ΔE^PNC^ for t_1/2_~one minute.

ΔE^PNC^ (au)	Selectivity Factor over 10^4^ Years	Selectivity Factor over 10^3^ Years	Selectivity Factor over 10^2^ Years	Selectivity Factor over 10 Years	Selectivity Factor over 1 Year	Selectivity Factor over 0.1 Year
10^−13^	1.74	1.06	1.01	1	1	1
10^−12^	253	1.74	1.06	1.01	1	1
10^−11^	1.1 × 10 ^24^	253	1.74	1.06	1.01	1
10^−10^	1.7 × 10 ^240^	1.1 × 10 ^24^	253	1.74	1.06	1.01
10^−9^	>10^300^	1.7 × 10 ^240^	1.1 × 10 ^24^	253	1.74	1.06

**Table 5:** Dependence of enantiomeric selectivities on ΔE^PNC^ for t_1/2_~one hour.

ΔE^PNC^ (au)	Selectivity Factor over 10^4^ Years	Selectivity Factor over 10^3^ Years	Selectivity Factor over 10^2^ Years	Selectivity Factor over 10 Years	Selectivity Factor over 1 Year	Selectivity Factor over 0.1 Year
10^−13^	1.01	1	1	1	1	1
10^−12^	1.10	1.01	1	1	1	1
10^−11^	2.51	1.10	1.01	1	1	1
10^−10^	1 × 10^4^	2.51	1.10	1.01	1	1
10^−9^	1.1 × 10^40^	1 × 10^4^	2.51	1.10	1.01	1

**Table 6:** Calculated ΔE^PNC^ values based on a metal-catalyzed Strecker reaction via a calcium-bound oxygen intermediate. ΔE^PNC^ values determined for single Ca^2+^, Sr^2+^, and Ba^2+^ centers, accounting for variations in orbital energies and atomic numbers. For Ca^2+^, Sr^2+^, and Ba^2+^, n′ = 4 to 8 orbital contributions, respectively, were considered for the parity term (P) in Equation (4), and the relative contributions from n′′ = 3 to 12 accounted for in the carbon spin-orbit coupling term (Δ) in Equation (5). Both conservative linear and angular arrangements are represented.

Cation	ΔE^PNC^ (au) (Angular)	ΔE^PNC^ (au) (Linear)	ΔE^PNC^ (au) (Linear) ALDOL [1]
_20_Ca^2+^	1.4 × 10^−12^	1.1 × 10^−14^	0.3 × 10^−20^
_38_Sr^2+^	3.2 × 10^−11^	7.6 × 10^−13^	0.6 × 10^−19^
_56_Ba^2+^	1.9 × 10^−10^	4.3 × 10^−12^	0.3 × 10^−18^

## Data Availability

No new data were created or analyzed in this study. Results of calculations are detailed in the manuscript.
